# Adaptation of the Th-MYCN Mouse Model of Neuroblastoma for Evaluation of Disseminated Disease

**DOI:** 10.3390/ijms241512071

**Published:** 2023-07-28

**Authors:** Seyed M. Rahavi, Maryam Aletaha, Ali Farrokhi, Amanda Lorentzian, Philipp F. Lange, Christopher A. Maxwell, Chinten James Lim, Gregor S. D. Reid

**Affiliations:** 1 Michael Cuccione Childhood Cancer Research Program, BC Children’s Hospital Research Institute, Vancouver, BC V5Z 4H4, Canada; 2 Department of Pediatrics, University of British Columbia, 950 W28th Avenue, Vancouver, BC V5Z 4H4, Canada; 3 Department of Pathology, University of British Columbia, 950 W28th Avenue, Vancouver, BC V5Z 4H4, Canada

**Keywords:** neuroblastoma, Th-MYCN, transgenic mouse, bioluminescence, transplantation, GD2

## Abstract

High-risk neuroblastoma remains a profound clinical challenge that requires eradication of neuroblastoma cells from a variety of organ sites, including bone marrow, liver, and CNS, to achieve a cure. While preclinical modeling is a powerful tool for the development of novel cancer therapies, the lack of widely available models of metastatic neuroblastoma represents a significant barrier to the development of effective treatment strategies. To address this need, we report a novel luciferase-expressing derivative of the widely used Th-MYCN mouse. While our model recapitulates the non-metastatic neuroblastoma development seen in the parental transgenic strain, transplantation of primary tumor cells from disease-bearing mice enables longitudinal monitoring of neuroblastoma growth at distinct sites in immune-deficient or immune-competent recipients. The transplanted tumors retain GD2 expression through many rounds of serial transplantation and are sensitive to GD2-targeted immune therapy. With more diverse tissue localization than is seen with human cell line-derived xenografts, this novel model for high-risk neuroblastoma could contribute to the optimization of immune-based treatments for this deadly disease.

## 1. Introduction

Neuroblastoma (NB) is one of the most frequently diagnosed embryonal cancers. With a median age at diagnosis of 17 months, NB is the most common cancer in infants and the third most common in children [1]. Clinical presentation ranges from an asymptomatic, localized tumor to widely metastasized, life-threatening disease [2]. NB treatment is based on risk, which is stratified by features such as stage, ploidy, MYCN amplification, and segmental chromosomal alterations [3]. Almost 50% of children who present with high-risk disease require multimodal therapy, which can include time-intensive chemotherapy [4], surgery, radiation, and stem cell transplantation [5]. Despite this aggressive treatment approach, the survival rate for children with high-risk NB remains only ~50%. Although retinoids and immunotherapies targeting GD2, as well as small molecule inhibitors targeting anaplastic lymphoma kinase (ALK) for the ~10% of patients harboring an *ALK* mutation, have emerged as valuable maintenance therapy options [6,7,8,9], new strategies are urgently needed to improve outcomes for the patients that subsequently relapse.

NB is a sympathetic nervous system tumor derived from the neural crest [2]. Almost half of cases present in the abdomen, mostly in the adrenal medulla, and the rest in sympathetic ganglia in paraspinal locations, including in the abdomen, chest and pelvis [2]. NB metastasizes to bone marrow, bone, liver, and lymph nodes in almost 70% of patients [10,11], with ~20% of patients presenting with two metastatic sites [11]. In some cases, NB can also metastasize to the brain, skin, and lungs [11,12,13,14]. Both lymphatic and blood circulation are involved in spreading NB cells [15,16]. To be effective for patients with the most clinical need, therapies must achieve eradication of NB cells from each of the diverse metastatic sites.

With a small patient population, the availability of experimental platforms for the rigorous preclinical screening of new agents for NB could greatly enhance therapy development. Both transgenic mouse models and mouse NB xenografts derived from cell lines (CDX) or patient tissue (PDX) are used in preclinical treatment development [17,18,19,20]. The Th-MYCN transgenic mouse, the most widely reported transgenic model of neuroblastoma, recapitulates many features of clinical NB progression and has been an important tool for the investigation of NB biology [21,22,23,24]. However, NB arising in Th-MYCN mice is a localized disease, and the model shows little propensity for metastasis [25]. To establish a platform for the testing of anti-NB activity across a broader range of tumor sites in an immune-competent setting, we developed a transplantation model utilizing primary luciferase-tagged NB cells isolated from tumors arising in Th-MYCN mice [21,26]. Localization of NB tumors to almost all clinically relevant sites was achieved in our cohort of recipient mice, providing more diversity of organ involvement than is observed in human NB CDX. This platform could contribute significantly to the optimization of immune-based treatment approaches for the eradication of NB at multiple sites.

## 2. Results

### 2.1. Bioluminescence-Based Monitoring of Neuroblastoma Progression in Th-MYCN Mice

**Figure 1:**
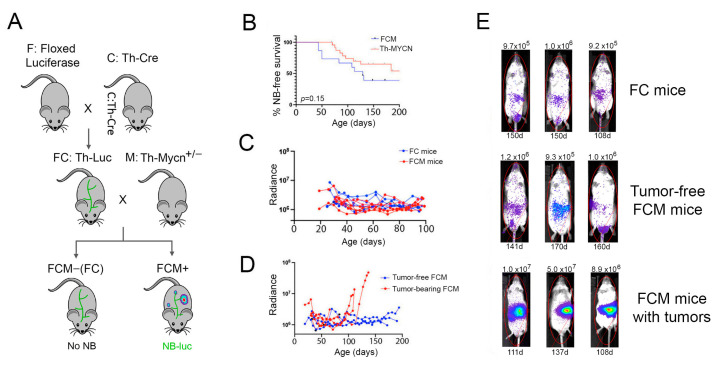
In situ development of luciferase-tagged neuroblastoma in FCM mice. (**A**) Schematic to show the triple transgenic breeding strategy for generating FCM mice that generate luciferase-expressing NB and control FC mice that show background luciferase expression in catecholaminergic neurons. (**B**) Penetrance of neuroblastoma in the FCM strain (24 mice) was similar to that in the parental Th-MYCN colony (15 mice), Log-rank *p* = 0.15. (**C**) Both Th-MYCN-positive FCM and negative (FC) mice show fluctuations in bioluminescent signal over the first 100 days of life. (**D**) NB development becomes clearly detectable above basal radiance in FCM mice at later stages of tumor progression. (**E**) Representative images of mice presented in panels C and D show the clarity of the NB-derived radiance signal in tumor-bearing FCM mice. The signal intensity and age (in days) at final image are shown at top and bottom of each image, respectively.

As the early stages of cancer progression in Th-MYCN mice can be challenging to detect, we first wanted to develop a more trackable model. To achieve this, we adopted a triple-transgenic approach to achieve luciferase expression in developing NB tumors (Figure 1A). Cre reporter mice carrying a floxed firefly luciferase gene (F) were crossed with mice bearing the Cre recombinase gene under control of the rat Th promoter (C), which is active in catecholaminergic neurons [27]. Progeny were then crossed to establish a colony of 129X1 mice that were homozygous for both transgenes (FC mice). Female FC mice were then bred with Th-MYCN hemizygous (M) males (also on 129X1 background) to generate progeny that either carried all three transgenes (called FCM mice) and were prone to NB development, or that lacked the MYCN transgene (FC mice) and, therefore, did not develop disease. The penetrance of neuroblastoma in Th-MYCN^+/−^ FCM mice matches that observed in our parental Th-MYCN colony (Figure 1B), and is comparable to that previously reported for MYCN transgene hemizygotes on the 129X1 background [22].

FCM mice enable monitoring of NB progression, or regression, longitudinally over the life of the mouse using bioluminescent imaging (BLI). However, both MYCN-positive (FCM) and MYCN-negative (FC) mice also express luciferase in non-malignant central and peripheral nervous system cells that at one stage during their development had an active Th promoter (and thus transiently expressed the Cre recombinase). Detection of developing NB tumors, therefore, requires the tumor-derived bioluminescent signal to rise above the background of luciferase activity in non-tumor-bearing mice. To assess whether early neuroblast hyperplasia driven by the MYCN transgene could be detected in FCM mice, we compared bioluminescent signals between cohorts of FC and FCM littermates from weaning until 100 days of age (Figure 1C). A range of baseline radiance was detected in both cohorts, with a general decline in signal to day 30 followed by fluctuations in individual FC and FCM mice over the remainder of the study. The absence of a clearly elevated signal in young FCM mice indicates that the early neuroblast dysplasia observed in Th-MYCN mice was below the level of detection of our luciferase-based reporter system [28].

In contrast to early hyperplasia, the later emergence of NB tumors in FCM mice was detectable as a persistent and increasing radiance signal of >5 × 10^6^, with the general localization and growth rate of developing tumors apparent weeks before humane endpoints were reached (Figure 1D,E). The bioluminescent signal in healthy adult FCM mice remained similar to that detected in FC mice (Figure 1E), indicating that tumor development was the sole driver of the increased radiance signal in older FCM mice. No tumors undetected by BLI were discovered during necropsy of euthanized FCM mice. Consistent with studies using the parental Th-MYCN strain, tumors in FCM mice arose in paraspinal locations, mostly in the abdomen, but occasionally in thoracic and pelvic regions [21,25]; tumors were not detected at sites associated with NB metastasis, such as bone marrow or liver, in any FCM mice.

### 2.2. Tagged Th-MYCN-Derived Tumors Are Serially Transplantable

**Figure 2:**
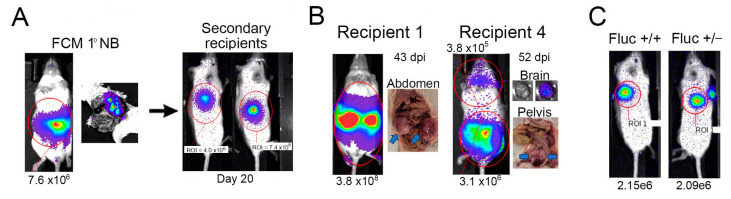
FCM-derived tumors are transplantable. (**A**) Primary NB tumor cells isolated from FCM mice are amenable to subcutaneous engraftment and rapid expansion in secondary recipients. (**B**) Examples of NB localization after intravenous injection of primary FCM tumor cells into immune-deficient recipients. Tumor location is indicated by arrows in non-BLI images. (**C**) Similar signal intensity was detected from 1 × 10^6^ Fluc^+/+^ and Fluc^+/−^ NB cells implanted on opposite flanks of a secondary recipient mouse. Radiance values are shown for the indicated regions of interest.

To assess the utility of FCM-derived NB cells for transplantation studies, we first injected 1 × 10^6^ tumor cells subcutaneously into immune-deficient recipient mice (Figure 2A). Rapid tumor growth was detected, reaching defined endpoint dimensions (1 cm^3^) within 4 weeks. We subsequently IV-injected these expanded FCM-derived NB cells into eight immune-deficient mice. Five of these recipients developed detectable tumors, with a range of time to endpoint of 43–96 days post-injection (dpi). While signal intensity detected from tumors in transplanted mice was comparable to that observed in primary FCM mice, the pattern of organ involvement was more diverse, with NB cells detected in cranium (two mice) and genital region (one mouse), in addition to abdominal and pelvic paraspinal locations (Figure 2B). These results support the utility of luciferase-tagged primary NB cell transplantation for the evaluation of more broadly disseminated Th-MYCN-derived tumors. However, signal intensity from even large tumors in transplanted mice did not exceed 5 × 10^8^ photons/s/cm^2^, indicating that luciferase expression is relatively low in FCM-derived NB cells (Figure 2B). An attempt to enhance this signal by generating FCM mice that carried an additional copy of the floxed luciferase gene was unsuccessful; the radiance emitted by equal numbers of NB cells with one or two copies of the luciferase gene was comparable (Figure 2C).

### 2.3. Transplanted Th-MYCN Tumor Cells Establish Broadly Disseminated Tumors

**Figure 3:**
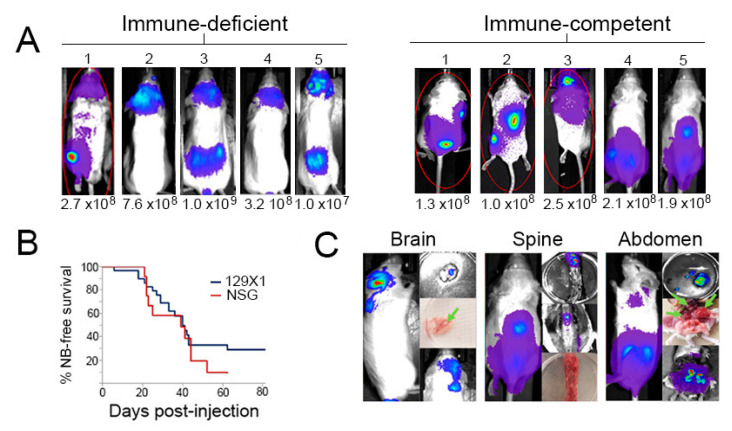
Localization and progression of luciferase-expressing Th-MYCN tumor cells. (**A**) Representative mice showing tumor detection following intravenous injection of 1–10 × 10^6^ lentivirus-transduced NB cells into NSG (immune-deficient) or 129X1 (immune-competent) recipients. (**B**) NB-free survival of immune-deficient (*n* = 9) and immune-competent (*n* = 15) recipients of primary, luciferase-transduced Th-MYCN NB cells. (**C**) Signal detection from isolated organs from mice at necropsy confirmed the source of radiance. Green arrows indicate the location of gross tumor.

Intravenous injection of tumor cells is often used as a low-barrier method to mimic the metastatic process and establish tumors at disseminated sites [29]. Our FCM mouse study established the potential for transplantation of tagged, primary NB cells to enable evaluation of tumor responses at multiple sites. However, the relatively low signal from FCM-derived tumors may limit the magnitude of response that can be detected. To enhance the resolution of detection of transplanted primary NB cells, we evaluated a second approach in which NB cells isolated from Th-MYCN tumors were transduced with a lentivirus vector containing genes for luciferase and GFP under the control of a strong promoter [30]. IV injection of transduced NB cells into immune-deficient (NSG or W41) or immune-competent (129X1) mice generated a pattern of localization similar to that observed with FCM-derived NB cells, but with generally elevated bioluminescent signals (Table 1 and Figure 3A) compared to FCM-derived NB tumors. 

Similar engraftment efficiency and progression kinetics were achieved in immune-deficient and immune-competent recipient mice, although about a quarter of 129X1 recipients did not engraft (Figure 3B). There was considerable overlap in the specific organ involvement observed in both types of recipient (Table 1). In both settings, the bioluminescent signal was frequently detected in the cranium (Figure 3A). Localizations to bone marrow, lung, kidney, brain, and skin were observed in at least one recipient (Table 1). Tumor locations were confirmed at necropsy by isolation and imaging of dissected tissues (Figure 3C). Importantly, these results indicate that, despite the high expression of the non-self GFP and luciferase proteins, tagged NB cells could establish tumors in fully immune-competent recipients. This approach thus provides a novel preclinical model for interrogating the contribution of immune mechanisms to the systemic or site-specific control of NB.

### 2.4. GD2-Targeted Depletion of Transplanted Th-MYCN Neuroblastoma

**Figure 4:**
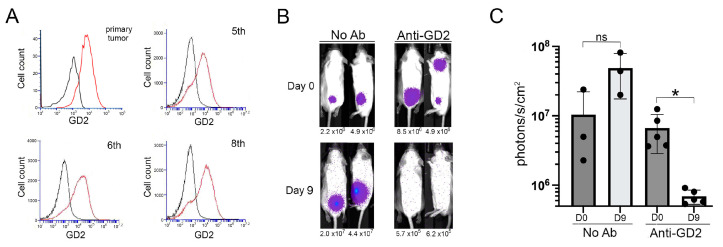
Targeting GD2 on transplanted Th-MYCN tumors. (**A**) GD2 expression on the source NB cells (primary tumor) and serially transplanted derivative cells (passage # indicated). Black lines = isotype control; red line = 14G2a antibody. (**B**) NB tumor imaging of control (No Ab) and treated (Anti-GD2) IV-injected 129X1 mice prior to (day 0) and after (day 9) completion of monoclonal antibody treatment. Whole animal signal intensities are shown for views with strongest signal at day 0. Mice from one representative experiment are shown. (**C**) Change in NB tumor burden between initiation (D0) and completion (D9) in control (*n* = 3) and Ab-treated (*n* = 5) mice. The y-axis begins at 5 × 10^5^, the average radiance for non-tumor bearing control mice. (Bars represent mean ± SD; * *p* < 0.05, ns = not significant; paired *t*-test).

Low GD2 expression on NB cell lines derived from Th-MYCN mice has limited the application of this model for development and optimization of GD2-targeted immune therapies, an area of significant clinical potential [8,9,31,32]. Notably, GD2 expression levels were maintained at levels comparable to that detected on the original primary Th-MYCN tumor through many rounds of in vivo expansion through various recipient strains (Figure 4A). This result indicates that lineage switching is not induced by the repeated disruption of tumor architecture needed to obtain single cell suspensions for re-injection [32]. 

To assess if this sustained GD2 expression rendered transplanted NB cells sensitive to targeted immune-mediated depletion, we treated 129X1 mice injected with NB-luciferase cells (10th passage) with the anti-GD2 monoclonal antibody, 14G2a. While tumor expansion was observed in untreated control mice (*p* = 0.08), there was a significant reduction of systemic bioluminescent signal in antibody-treated mice within 9 days of treatment initiation (*p* = 0.02, Figure 4B,C). This result demonstrates the utility of the Th-MYCN transplant model for the assessment of targeted immune therapies at disparate sites. 

### 2.5. Localization and Growth in Human NB Cell Lines in Xenografts

**Figure 5:**
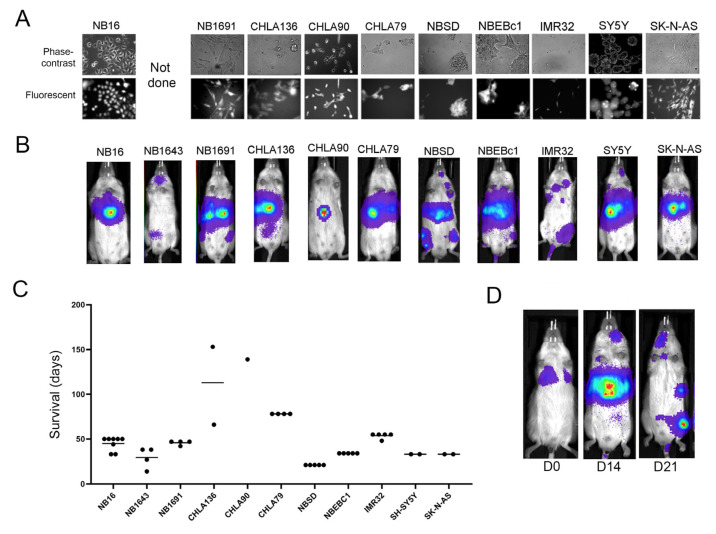
Localization of IV-injected human neuroblastoma cell lines. (**A**) Phase-contrast and fluorescent images of human NB cell lines confirmed successful GFP expression after transduction. (**B**) Images of representative NSG xenografts for each cell line after IV injection. (**C**) Rates of tumor progression for human cell line-derived NSG xenografts. Each data point respresents a recipient mouse. (**D**) NB redistribution observed in NSG mice injected with NBSD cells.

Human NB xenografts in immune-deficient mice have been used extensively for preclinical treatment evaluation. To investigate if the diverse localization of Th-MYCN NB cells similarly transplanted into NSG mice matched that obtained with human NB transplant models, we developed a panel of cell line-derived xenografts that reflected some of the genomic heterogeneity of NB (Table 2). Using the same GFP/luciferase-containing lentivirus construct as was used for Th-MYCN tumors, we established 11 stably tagged human NB cell lines (Figure 5A). Following intravenous injection of 0.5–1 × 10^6^ cells, each cell line displayed generally consistent engraftment, outgrowth kinetics, and localization patterns in co-recipient mice (Table 3, Figure 5B,C). In contrast to primary Th-MYCN-derived NB cells, localization to the liver was detected with all human NB cell lines. Five of the lines also showed bone marrow involvement in at least half of the recipient mice. Cranial involvement was only detected in NBSD recipients and one recipient of the NB1643 cell line. Abdominal masses were rarely seen with the human NB cells. In general, involved sites in individual mice were consistent from first detection through to endpoint. However, a loss of signal from the liver coincident with an increase in bone marrow was observed in some, but not all, NBSD recipients in multiple experiments (Figure 5D). This fluctuation was unique to NBSD recipients in our study.

## 3. Discussion

High-risk NB patients are in dire need of new treatment options. Targeted therapies, both in the form of precision drugs and immunotherapy, offer significant potential to improve outcomes. Optimization of these therapies, however, will require rigorous assessment of potential combination approaches to achieve durable protection from disease progression. As the tumor niche represents an important biological variable [20,33,34], here we report an immune-competent Th-MYCN-based mouse model that could provide a platform for the in-depth evaluation of localized anti-NB therapeutic activity, including the contribution of diversified immune responses generated downstream of drug, monoclonal antibody, or adoptive cell transfer interventions. Both primary and transplanted luciferase-tagged Th-MYCN tumors are readily detectable by bioluminescent imaging, allowing assessment of disease progression at diverse sites. 

Notably, a distinct pattern of tumor localization was observed between human cell lines and primary mouse NB cells, with liver involvement only observed with the human lines. Transplanted Th-MYCN cells showed broader tissue involvement, but with greater variation between recipients. In contrast, human NB cell lines generated a more restricted pattern of tissue localization and higher degree of reproducibility. As alternative injection strategies have been shown to expand the array of metastatic sites in human NB xenografts [35,36], it will be of considerable interest to determine if such approaches can be used to further improve the Th-MYCN transplant model by increasing reproducibility, while maintaining the diversity of tissue involvement. However, even with tail vein injection, cohorts of mice receiving Th-MYCN-derived primary NB cells will enable the assessment of tumor responses at multiple clinically relevant tissue sites.

The Th-MYCN mouse has been an integral component of NB research for a quarter century [26]. During that time, however, its application for immune studies has been limited. This has resulted, in large part, from the failure of cell lines derived from Th-MYCN tumors to express the GD2 antigen, which has been the focus of the majority of immunotherapies for NB [31,32]. The recent demonstration that the GD2 expression is present on primary Th-MYCN tumors, but lost on cell lines as a result of lineage switching in vitro, has provided support for the use of this strain in immune studies [32,34]. Importantly, our study reveals that GD2 expression on primary Th-MYCN tumor cells is stable through multiple rounds of engraftment and expansion, and provides proof-of-principle that transplant studies could facilitate detailed investigations of anti-NB immune activity and long-term protection. 

The application of human NB xenografts has been useful for treatment evaluations [37,38,39]. Although recipient mice are profoundly immune-deficient, these models are amenable to assessment of some immune interventions, such as adoptive cell therapy [40,41]. However, secondary immune activities cannot be assessed in such models. In this regard, the immune-competent Th-MYCN transplant studies described here could enable a deeper assessment of immune-mediated determinants of durable NB control. The combined use of both syngeneic and xenografted models likely represents the optimal testing strategy, with the ability to assess the efficacy of both direct human NB cell killing in immune-deficient recipients and the impact of downstream activities, such as epitope spreading, on durable protection in immune-competent hosts. The platforms described in this study could have immediate application in this emerging area. 

Overall, our study expands on the recent demonstration of GD2 expression by Th-MYCN-derived NB tumors to demonstrate the utility of this model for assessment of anti-NB activity in cohorts of recipient mice. This approach could increase throughput, with better control of experimental variables, and reduce the number of mice required for in-depth investigation of protection against NB progression. Such a model could contribute significantly to the identification of combination therapies that achieve tumor eradication and long-term immune protection.

## 4. Materials and Methods

### 4.1. Cell Lines

Several well-characterized human NB cell lines were generously provided by Drs. David Barrett (University of Pennsylvania, Philadelphia, PA, USA), Patrick Reynolds (Texas Tech), and Peter Houghton (University of Texas, San Antonio, TX, USA) [42,43,44,45,46]. Cells were cultured in standard medium (Eagles Modified, DMEM, or RPMI; Thermo Fisher Scientific, Waltham, MA, USA) supplemented with 10% fetal bovine serum, penicillin/streptomycin, 1% L-glutamine, 2% Gibco HEPES buffer, 1% non-essential amino acids, and 0.02% 2-mercaptoethanol (all media components from Thermo Fisher Scientific). The previously reported genomic lesions in each cell line were confirmed by targeted sequencing (Table 2). Briefly, library preparation and targeted sequencing using the Oncomine Childhood Cancer Research Assay (OCCRA, Thermo Fisher Scientific) were performed on Ion Chef and Ion Torrent S5 platforms (Thermo Fisher Scientific) following the manufacturer’s protocols. The average read depth for the OCCRA panel was 5–7 × 10^6^ per sample for DNA and 1–2 × 10^6^ for RNA. SNVs were retrieved with Ion Reporter software (version 5.2). Copy number measurements were retrieved with Ion Reporter software (version 5.2) for genes with >5 probes, including those that were validated for copy number gains as described elsewhere [47].

### 4.2. Mice

129X1/SvJ-Tg(TH-MYCN)41Waw/Nci mice (Th-MYCN), which express the human MYCN gene under control of the rat tyrosine hydroxylase (Th) promoter, were re-derived from sperm obtained from the NCI National Laboratory (Frederick, MD, USA). A colony of Th-MYCN hemizygotic mice was maintained by in-house breeding. Males and females were used for all experiments. Wild-type syngeneic 129X1 mice were generated for experiments by mating of Th-MYCN transgene-negative littermates. B6.Cg-7630403G23Rik mice expressing a Cre recombinase under control of the Th promoter, and firefly luciferase-based Cre reporter FVB.129S6(B6)-Gt(ROSA)26Sortm1 mice (Fluc) were purchased from The Jackson Laboratory (Bar Harbor, ME, USA) and bred onto the 129X1 background for seven generations. Together with Th-MYCN mice, these strains were used to generate a novel Fluc/Cre/MYCN (FCM) triple transgenic line, which was maintained as Fluc- and Cre-homozygotes, and MYCN-hemizygotes by sibling mating. Immune-deficient NOD.Cg-Prkdcscid/IL2rgtm1Wjl/SzJ (NSG) mice (Jackson Laboratory, Bar Harbor, ME, USA) and NOD.Cg-*Rag1^tm1Mom^ Kit^W−41J^ Il2rg^tm1Wjl^*/EavJ mice (W41; generously provided by Dr. Connie Eaves, University of British Columbia) were maintained by in-house breeding. This study was approved by the University of British Columbia (UBC) Animal Care Committee. All experiments were performed in accordance with the relevant guidelines and regulations pertaining to our UBC-approved protocol (A19-0197). 

### 4.3. Isolation of Primary Th-MYCN-Derived NB Cells

Progression of NB in Th-MYCN mice was assessed by twice-weekly visual inspection and abdominal palpation. Upon detection of impaired movement or a palpable mass, mice were euthanized, and the tumors extracted. Tumors were cut into small pieces in ice cold high-glucose DMEM media with DNase, and the supernatant removed after sitting for 1 min. This process was repeated 3 times, then the pooled supernatants were passed through a 40 µm cell strainer, then centrifuged for 3 min at 200× *g*. The cell pellet was re-suspended in 1 mL high-glucose DMEM with 10% FBS and 2-mercaptoethanol and viable cells counted. 

### 4.4. Luciferase-Tagging Neuroblastoma Cells

Lentiviral constructs driving expression of click beetle green (CBG) or click beetle red (CBR) luciferases and green fluorescent protein (GFP) (pELNS.CBG-T2A-GFP and pELNS.CBR-T2A-GFP, respectively) were generously provided by Dr. David Barrett (Uni. of Pennsylvania) [30]. For human NB cell lines, 1 × 10^5^ cells were placed in the center of a well in a 6-well plate, forming an undisturbed half-round drop. After incubating for 20 min to allow cells to adhere to the base of the well, cells were washed, and 50 µL of FBS-free media containing the lentivirus was added dropwise on top of the cells. The plate was incubated for one hour, then 150 µL of media was added to the center drop, and incubation continued overnight. Cells were then washed, and the well was filled with 5 mL of appropriate media. After a further 24 h, 2 µL of 1% D-Luciferin was added to the media, and cells were imaged to confirm bioluminescent signal. For primary Th-MYCN tumor cells, a modified protocol was used due to the high death rate observed during conventional transduction. Briefly, 2 × 10^6^ pelleted cells were resuspended in 14 µL of concentrated lentivirus, and the mixture incubated for 5 min at 37 °C. Then, 200 µL of high glucose DMEM was added, and incubation continued for 2 h. The cells were washed four times with high-glucose DMEM, resuspended in matrigel, and implanted via subcutaneous injection into the flanks of NSG mice for in vivo expansion. Transduced human and mouse NB cells, after in vitro or in vivo expansion respectively, were enriched for GFP expression by two rounds of flow cytometry-based sorting, and cryopreserved for future use.

### 4.5. Longitudinal Monitoring of NB Growth

For tumor transplantation experiments, 0.1–1 × 10^6^ luciferase-tagged NB cells were engrafted via intravenous (IV) or subcutaneous (SQ) injection. Transplanted or in situ developing NB tumors were imaged on an Ami-X platform (Spectral Instruments Imaging, Tucson, AZ, USA) and analyzed using AMIView. Mice were intraperitoneally injected with a 1% solution of D-Luciferin Potassium Salt (Goldbio, St. Louis, MO, USA) in PBS 8 min prior to in vivo imaging. All images from individual experiments are presented on the same scale, and radiance from the indicated regions of interest is provided. Where indicated, mice were administered up to six 100 mg doses over 14 days of the anti-GD2 monoclonal antibody, 14G2a (BioXcell, Lebanon, NH, USA), and monitored for NB growth or regression.

### 4.6. Statistical Methods

Kaplan–Meier curves generated for NB transplantation and in situ survival studies were analyzed by log-rank tests. Statistical analyses were performed using Prism 5 for Mac OS X (GraphPad, San Diego, CA, USA). *n* values for each experiment are listed in figure legends. 

## 5. Conclusions

There is an urgent need for improved treatment options for patients with high-risk NB. Precision medicine and immunotherapy both show significant potential to address this need, but an increased availability of preclinical models that recapitulate aspects of the human disease is required to enable the rapid and rigorous assessment of emerging approaches. The transplantation-based approach, utilizing luciferase-tagged, primary Th-MYCN NB cells, developed in this study overcomes previous limitations of this model for the evaluation of NB growth and regression at secondary sites. The approach consistently generates a more broadly disseminated disease than human NB cell line xenografts, and can provide a useful platform for the evaluation of treatment responses in fully immune-competent, as well as specifically or globally immune-deficient, settings.

## Figures and Tables

**Table 1:** NB engraftment and localization in secondary recipients.

Immune Status	Injected	Engrafted	Survival (Days)	Paraspinal	BM	Cranial	Other
Competent	15	11	41 ± 28	5	4	9	3
Deficient	9	8	43 ± 24	2	4	7	1

**Table 2:** Human NB cell line genomics.

	MYCN	ALK	TP53	Other SNV
CHLA90		F1245V	TP53 E286K	CIC
NB1643	Amplified	R1275Q	WT	
NBSD	Amplified	F1174L	TP53 C176F	ARID1A, DICER
SK-N-AS		WT	CNV 0.5	NRAS
NB16		WT	TP53 R248W	PIK3CA, NF1, NRAS
SY5Y		F1174L	WT	SMARCA4
CHLA79	Low-amplified (4.9)	WT	WT	
CHLA136	Amplified	WT	WT	
NB1691	Amplified	WT	WT	
NBEBc1	Low-amplified (3.8)	WT	WT	KRAS
IMR32	Amplified	WT	WT	

**Table 3:** Human cell line localization.

Cell Line	Liver	BM	Cranial	Other
CHLA90	1/6			
NB1691	4/4	2/4		
NBSD	5/5	7/7	6/7	
SK-N-AS	3/3			
NB16	9/9			1/9 kidney
SY5Y	5/5			
CHLA79	4/4	2/4		
CHLA136	2/4			1/4 abdominal
NB1643	2/4		1/4	3/4 abdominal
NBEBc1	5/5	3/5		
IMR32	5/5	5/5		

## Data Availability

Not applicable.
